# Stem cell-derived extracellular vesicle-based therapy for nerve injury: A review of the molecular mechanisms

**DOI:** 10.1016/j.wnsx.2023.100201

**Published:** 2023-04-25

**Authors:** Davood Nasiry, Ali Reza Khalatbary

**Affiliations:** a Amol Faculty of Paramedicine, Mazandaran University of Medical Sciences, Sari, Iran; b Cellular and Molecular Research Center, Mazandaran University of Medical Sciences, Sari, Iran

**Keywords:** Stem cell, Extracellular vesicle, Nerve injury, Clinical trial, *In vivo*, *In vitro*

## Abstract

Recent evidence suggests that stem cell therapy has beneficial effects on nerve damage. These beneficial effects were subsequently found to be exerted in part in a paracrine manner by the release of extracellular vesicles. Stem cell-secreted extracellular vesicles have shown great potential to reduce inflammation and apoptosis, optimize the function of Schwann cells, regulate genes related to regeneration, and improve behavioral performance after nerve damage. This review summarizes the current knowledge on the effect of stem cell-derived extracellular vesicles on neuroprotection and regeneration along with their molecular mechanisms after nerve damage.

## 1 Introduction

Functional impairment and long-term disability caused by nerve injury are among the major clinical and public health problems.^1^ According to severity, nerve injury is classified into three main groups called neurapraxia with localized myelin degeneration and without axonal degeneration, axonotmesis with complete axonal interruption resulting Wallerian degeneration, and neurotmesis with complete nerve interruption.^2^ Among the factors affecting the improvement of nerve function after injury are the location and degree of nerve damage and factors related to the patient.^3^ In this regard, there are generally three accepted repair mechanisms, including Schwann cell-mediated remyelination, axonal regrowth, and collateral sprouting.^4^ Presently, clinical treatments for nerve injury mainly include end-to-end repair with advanced microsurgery surgery along with postoperative rehabilitation. However, peripheral nerve repair often does not lead to satisfactory functional improvement, and it is clear that the methods of the microsurgery are not very effective against the complex molecular and cellular events caused by nerve damage. Accordingly, some therapeutic strategies have been mentioned to accelerate the regeneration of axons following injury such as pharmacotherapy, electrical stimulation, hyperbaric oxygen therapy, and cell-based therapy.^5^, ^6^, ^7^, ^8^, ^9^ It is well known that Schwann cells provide a favorable and facilitating environment for the growth of the damaged nerve and play a critical role in degeneration, remyelination and axonal growth of peripheral nervous system.^4^^,^^7^ Since the absence of healthy Schwann cells after severe nerve damage is one of the key points in nerve regeneration,^10^^,^^11^ the idea of replacing Schwann cells through cell-based therapy has been considered. In this regard, the use of Schwann cell culture has shown desirable results in the experimental nerve injury model through regeneration and remyelination. However, since the process of collecting human Schwann cells is an invasive method and the potential for proliferation of these cells *in vitro* is limited,^12^ researchers have been looking for other suitable Schwann cell sources. Among these sources, stem cells have received much attention due to their availability, *in vitro* rapid proliferation, integration, and survival into the host tissue. So far, stem cells with different origins and types have been used for *in vitro* Schwann-like cell generation and cell-replacement therapy, including embryonic stem cells (ESCs), mesenchymal stem cells (MSCs), induced pluripotent stem cells (iPSC), and neural stem cells (NSCs).^7^^,^^13^ Regarding the regenerative effects of cell therapy, mechanisms such as the replacement of damaged endogenous Schwann cells have been proposed, which accelerates the regeneration and remyelination of the injured nerve.^14^^,^^15^ Another possible mechanism is the production of neurotrophic factors, including ciliary-derived neurotrophic factor (CDNF), nerve growthe factor (NGF), brain-drived neurotrophic factor (BDNF), glial cell-drived neurotrophic factor (GDNF), and neurotrophin-3 (NT-3), which facilitate the regeneration of injured nerve.^16^, ^17^, ^18^ Recent evidence supports stem cell-mediated protection and repair of tissue through the paracrine signaling pathway.^19^^,^^20^ Subsequently, it was found that the benefits may be due to the release of extracellular vesicles derived from stem cells into the conditioned media (*in vitro*) or injection site (*in vivo*).^21^^,^^22^ Cell-derived membrane-bound vesicles, known as extracellular vesicles (EVs), are classified based on their size and origin as exosomes, apoptotic bodies, and microvesicles.^23^ Studies confirm the role of EVs derived from stem cells in neurodevelopment, neuroplasticity, neuroprotection, neuroregeneration, and improvement of neural dysfunction.^24^ On the other hand, using EVs derived from stem cells has several advantages compared to the cells themselves, including passing through the blood–brain barrier and subsequently penetrating into different parts of the nervous system following injection, no malignant transformation and tumorigenesis, no thrombogenicity, low immunogenicity, easy to store and transport, and easy to use through non-invasive administration methods.^25^, ^26^, ^27^, ^28^ Recently, experimental studies revealed that EVs can provide neuroprotective actions against some nervous system disorders such as nerve injury. The beneficial effects are mainly attributed to optimization of Schwann cell function,^29^ autophagy reduction,^30^ anti-inflammatory effects,^31^ anti-apoptotic effects,^32^ and regulation of regeneration-related genes.^33^

This study is focused on the neuroprotective and regenerative effects of stem cell-derived EVs along with the molecular mechanisms responsible for these properties following nerve injury.

## 2 Methods

The literature search was performed using PubMed and Scopus, supplemented with Google Scholar and reference lists of relevant articles based on articles published from 1951 to 2023. The key terms used (MeSH database) were “stem cell”, “extracellular vesicle”, “neural injury”, “clinical trial”, “*in vivo*”, and “*in vitro*".

### 2.1 *In vivo* studies

A growing number of *in vivo* experimental investigations on the protective and regenerative benefits of stem cell-derived EVs against neuronal injury along with their molecular mechanisms have begun to accumulate, which are listed in Table 1. Ma and colleagues^34^ for the first time investigated the beneficial effects of bone marrow mesenchymal stem cells (BMSCs)-derived EVs after local administration in crushed rat sciatic nerve. The results of this study revealed that the injection of EVs into the lesion site significantly promoted nerve regeneration and improved the sciatic function index (SFI) compared to the control rats. After that, Bucan et al^35^ compared the benefits of adipose-derived mesenchymal stem cells (adMSCs) with adMSCs-drived exosomes following nerve crush injury in rat. In this study, while increasing the rate of axonal regeneration in both experimental groups compared to the control group, the transplantation of exosomes compared to adMSCs caused more regeneration of nerve fibers. In addition, a significant improvement in the SFI was observed only in the exosome-treated animals compared to the control rats. Regarding the possible mechanism of action of exosomes, it has been proposed that exosomes accelerate the regeneration process of peripheral nerve through optimizing Schwann cell function.^29^ Olfactory ensheathing cell (OEC) is another known source of EVs for nerve regeneration. Xia et al^36^ for the first time showed that axon regeneration and myelination along with functional recovery were significantly increased in rat injured sciatic nerve using neural conduits containing OECs-EVs. It is well known that human umbilical cord mesenchymal stem cells (hUCMSCs) have stronger paracrine effects than adipose-derived mesenchymal stem cells or bone marrow stem cells.^37^ In this regard, it was documented that hUCMSC-EVs through down-regulation of interleukin (IL)-6 and IL-1β as well as up-regulation of IL- 10 improved functional recovery, accelerated nerve regeneration, and modulated muscle atrophy following rat sciatic nerve transaction.^31^ Allodynia and hyperalgesia are well-known features of neuropathic pain that can be caused by peripheral nerve injury.^38^ In this regard, Shiue et al^39^ used exosomes derived from hUCMSC as a cell-free treatment for pain caused by nerve damage following L5/6 rat spinal nerve ligation. The results showed that the injection of exosomes by intrathecal route reversed the thermal and mechanical hypersensitivities caused by nerve ligation in the early and advanced stages of pain. In addition, 2′,3′-cyclic nucleotide 3′-phosphodiesterase (CNPase), ionized calcium-binding adapter molecule 1 (Iba1), glial fibrillary acidic protein (GFAP), and c-Fos upregulation suppressed with exosome. Meanwhile, exosome treatment reduced the levels of tumor necrosis factor alpha (TNF-α) and IL-1β as well as increased the levels of glial cell-derived neurotrophic factor (GDNF), brain-derived neurotrophic factor (BDNF), and IL-10. Mao and colleagues^40^ investigated the extent of nerve regeneration by gingival-derived mesenchymal stem cells (GMSCs)-derived EVs in sciatic nerve-injured mice. Results of this study showed that topical gelfoam coating included with extracellular vesicles derived from GMSCs at the site of crush injury improved axonal regeneration and function, possibly through activating repair Schwann cell phenotype governed by transcription factor c-Jun, which is comparable with the effects of direct GMSCs transplantation. Another study showed that exosomes from human GMSCs combined with biodegradable chitin conduits significantly increase the number and diameter of nerve fibers and promote myelin formation following rat sciatic nerve transection.^41^ As previously mentioned, olfactory ensheathing cells are reliable and promising cell sources for axonal regeneration. Although, their utility is limited due to the hypoxia that usually exists at the lesion site. On the other hand, exosomes derived from human umbilical cord mesenchymal stem cells have the potential to regulate hypoxia-induced pathological processes. Results of a study showed that combination of exosomes derived from human umbilical cord mesenchymal stem cells and olfactory ensheathing cells can increase the olfactory ensheathing cell survival and migration in hypoxia (*in vitro*) and synergistically improved nerve regeneration as well as sensory and motor function of the damaged sciatic nerve (*in vivo*).^42^ In order to compare the regenerative effects of stem cell with their extracellular vesicles, Chen et al^43^ used human embryonic neural stem cells-derived microvesicles alongside these cells. Results of this study showed that a silicon tube filled with the microvesicles improves morphological recovery and increases the number of regenerated axons compared to the stem cells during 12 weeks after nerve injury. Locally applied an alginate scaffold with exosomes derived from human umbilical cord mesenchymal stem cell attenuated pain resulting from nerve injury in rats so that the rats had a higher withdrawal threshold and latency, possibly through decreased levels of IL-1β, GFAP, Iba1, TNF-α and c-Fos, and increased levels of IL-10, GDNF and myelin basic protein.^44^ Another study conducted to elucidate the molecular mechanisms participating in peripheral nerve repair using exosomes derived from bone marrow stromal cell.^33^ The results showed that exosomes derived from bone marrow stromal cells can enhance peripheral nerve regeneration and that the mechanism may engage VEGFA as a regeneration-related gene. Moreover, a dose-dependent relationship was seen between nerve regeneration and exosome therapy. As mentioned earlier, following peripheral nerve damage, exosomes derived from adipose-derived stem cells (ADSC-exo) enhance the damaged nerve regeneration. Two studies were conducted on the effect of ADSCs pretreatment with an immunosuppressive drug (FK506) on the release of exosomes (ADSC-F-exo) and possible enhancement of nerve regeneration in crushed mouse sciatic nerve.^45^^,^^46^ Results of these studies showed that locally applied ADSC-F-exo significantly increased nerve repair and reduced autophagy of macrophages but it could not secrete exosomes that have stronger molecules for nerve regeneration relative to ADSC-exo. It is well known that nanosized vesicles such as exosome can also be used as carriers of neuroprotective substances. One of the exosomal cargoes, microRNA (miRNA), downregulates genes associated with inflammation. Fan et al^47^ used miR-146a-loaded MSC-exosomes in treatment of a mouse diabetic peripheral neuropathy. This study found that diabetic peripheral neuropathy treatment with exo-146a significantly increased neurological recovery compared to MSC-derived exosomes (exo-naïve). Also, exo-146a significantly inhibited endothelial cell and monocyte activations through the Toll-like receptor (TLR)-4/NF-κB signaling pathway inhibition compared with exo-naïve. Schwann cells and autophagy play a critical role in the regeneration of the damaged nerve. A study investigated the effect and mechanism of adipose-derived stem cell-derived exosomes (ADSC-Exos) on autophagy of Schwann cells and myelin sheath regeneration after peripheral nerve injury.^30^ Overall, results of this study showed that ADSC-Exos improve myelin sheath regeneration by reduction of damaged Schwann cells autophagy through downregulating karyopherin alpha 2 subunit (Kpna2). Biomimetic nerve conduction conduits containing engineered adipose-derived stem cell exosomes have recently been used to accelerate the process of nerve regeneration.^48^ In this study, NT-3 mRNA encapsulated in the exosome (Exo^NT−3^) was loaded in nerve guidance conduit (Exo^NT−3^-NGC) to create a bridge in the damaged part of rat sciatic nerve. The results of this study indicated that ExoNT-3-NGC significantly accelerates the regeneration of the damaged nerve and improves gastrocnemius muscle function compared to the control. Li et al documented that intrathecally administration of the bone marrow mesenchymal stem cell-derived exosomes loaded with miR-150–5p alleviate mechanical allodynia through targeting neurogenic locus notch homolog protein 2 (NOTCH2) in microglial cells following L5 spinal nerve ligation model in rat.^49^ In this regard, another study showed that a single intrathecal injection of the human placental mesenchymal stem cells (hPMSCs)-derived small extracellular vesicles durably reversed mechanical hypersensitivity and neuroinflammation through miR-26a-5p/Wnt5a in mice with partial sciatic nerve ligation.^50^ Histological and functional evaluation of the crushed sciatic nerve after transplanting the gel containing human multipotent mesenchymal stromal cell-derived extracellular vesicles (MSCs-EVs) showed a significant decrease in apoptotic neuronal death and an increase in regeneration-associated proteins NF-200 and GAP-43, as well as a decrease in muscle atrophy.^51^ Exosomes from Lipopolysaccharide (LPS)-preconditioned mesenchymal stem cells accelerated functional recovery, axon regeneration and remyelination through proinflammatory macrophage-to-progenitor macrophage transition. Meanwhile, the investigators discovered the involvement of TSG-6/NF-κB/NLRP3 signaling pathway in this process by an inflammatory model in RAW264.7 cells.^52^ A study has shown that the expression of miR-181c-5p is clearly decreased in a time-dependent manner following nerve chronic constriction injury (CCI), so that intrathecal administration of exosomes carrying miR-181c-5p reduces neuropathic pain by inhibiting inflammatory factors secretion in rat sciatic nerve CCI.^53^ Chen et al documented that the thermosensitive hydrogel carrying extracellular vesicles from adipose-derived stem cells increases the diameter of the fascicle and myelin thickness, as well as increases the nerve conduction efficacy and contraction force of leg muscles innervated by the repaired nerve following microsurgical repair.^54^ Recently, it has been shown that reduced graphene oxide-embedded nerve conduits loaded with bone marrow mesenchymal stem cell-derived extracellular vesicles can improve neurological function recovery by increasing the number of newly formed vessels and axonal sprouts at the site of nerve injury.^55^ Interestingly, Zhu et al found that small extracellular vesicles produced from umbilical cord mesenchymal stem cells cultured under hypoxia accelerated Schwann cells recruitment at the site of nerve injury and enhanced nerve repair and regeneration in a mouse model of sciatic nerve injury; because in the condition of hypoxia, the amount of secreted small extracellular vesicles increases significantly compared to the control cells.^56^

**Table 1:** Summary of studies of the effects of EVs derived from stem cells on nerve injury.

Nerve injury models	Treatment programs	Findings	Involved mechanisms	References
Rat sciatic nerve crush injury	EVs (45 μg/30 μL) immediately injected into the lesion site using a microinjector	Improvement of the sciatic function index, promotion of the myelin and axon regeneration	Without providing possible mechanisms	34
Rat sciatic nerve crush injury	PKH-26-stained adMSC exosomes were administered with a Hamilton syringe into the distal and proximal regions of the injured site	Improvement of the sciatic function index, increased axonal regeneration (S100 and neurofilament)	Without providing possible mechanisms	35
Rat Sciatic nerve transection	MSCs-exosomes (100 μg protein estimated to be 2.5 × 109) injected in Saphenous vein	Improvement of axon myelination and regeneration, repair of denervated muscle atrophy	Optimizing Schwann cell function	29
Rat Sciatic nerve transection	OECs-EVs-Matrigel mixture (108 particles/ml) used with conduit	Improvement of the sciatic function index and nerve conduction velocity, enhanced myelination and axonal regeneration	Without providing possible mechanisms	36
Rat Sciatic nerve transection	Tail vein injection of 100 μg of hUCMSC-EVs after 24 h of injury induction	Up-regulation of IL-10 and down-regulation of IL-6 and IL-1β, improved functional recovery, increased myelinated fibers	Via regulation of the pro- and anti-inflammatory cytokines	31
Rat spinal nerve ligation	Injection of 10-mL UCMSC exosome (0.12, 0.6, and 1.2 mg/mL) into the intrathecal space by catheter	Reversed hypersensitivities, suppressed GFAP, Iba1, c-Fos and CNPase, inhibited IL-1β and TNF-α, enhanced GDNF, IL-10 and BDNF	Via anti-inflammatory and pro-neurotrophic abilities	39
Mice sciatic nerve crush injury	Injection of GMSC-derived EVs (40 μg) mixed with Gelfoam (4 × 4 × 3 mm) immediately after injury	Promoted axon regeneration (β-tubulin Ш and S-100 β) and functional recovery	Via activating Schwann cell phenotype regulated by c-Jun	40
Rat Sciatic nerve transection	Chitin conduit plus 10 μL PBS containing 10 μg exosomes	Increased diameter and number of nerve fibers, promoted myelination, recovered nerve and muscle function	Without providing possible mechanisms	41
Rat Sciatic nerve transection	Exosomes (400 μg/mL) and 10 μL OECs (5 × 10^5^) used with conduit	Promoted nerve regeneration, improved sensory and motor function	Without providing possible mechanisms	42
Rat Sciatic nerve transection	hESC–NSC–MVs (45 ​μg) diluted in 20 ​μl DMEM/F12+ Matrigel used with conduit	Greater regenerated axon number and better morphological recovery	Without providing possible mechanisms	43
Rat nerve ligation	Scaffold loaded with 25 μL exosome solution (1 mg/mL)	Attenuated pain, Iba1, TNF-α, c-Fos, GFAP and IL-1β, enhanced GDNF, IL-10 and myelin basic protein	Via pro-neurotrophic and anti-inflammatory properties	44
Rat sciatic nerve crush injury	Injection of 4 μl of exosomes in the proximal 2/10 and 3/10 of the calf length of gastrocnemius muscle	Increased regenerated axon number and diameter, improved latency of thermal pain and functional index, increased VEGFA, S100b, NGFr and PMP22 expression	Via regeneration-related genes regulation	33
Mice diabetic peripheral neuropathy	Tail vein injected of Exo-naïve and exo-146a were once a week for 4 consecutive weeks	Increased nerve conduction velocity, decreased mechanical and thermal stimuli threshold, suppressed the activation of endothelial cells and inflammatory monocytes in the peripheral blood	Through the Toll-like receptor (TLR)-4/NF-κB signaling pathway inhibition	47
Rat sciatic nerve injury	Not available	Promoted myelin sheath regeneration and reduced damaged Schwann cell autophagy	Via Kpna2 downregulation	30
Rat sciatic nerve injury	Exo^NT−3^-alginate hydrogel syringed into a 10-mm silicone tube as the external conduit	Promoted nerve regeneration and improved gastrocnemius muscle recovery	Restoration of the level of NT-3	48
L5 spinal nerve ligation model in rat	Intrathecally administration of the BMSC-derived exosomes loaded with miR-150–5p	Alleviated mechanical allodynia, reduced apoptosis and inflammation	Through targeting neurogenic locus notch homolog protein 2	49
Mice partial sciatic nerve ligation	A single intrathecal injection of the hPMSCs-derived extracellular vesicles on the seventh day of modeling	Reversed mechanical hypersensitivity in the hind paw and neuroinflammation	Through miR-26a-5p/Wnt5a	50
Rat sciatic nerve crush injury	The collagen-gel-based substance was applied in 1 h after nerve crushing to the damaged nerve	Reduced muscle atrophy and apoptotic neuronal death and increased NF-200 and GAP-43 in DRG and damaged nerve	Without providing possible mechanisms	51
Rat sciatic nerve injury	Local injection of LPS pre-Exos into the nerve injury site	Accelerated functional recovery, axon regeneration and remyelination, shifted the pro-inflammation macrophage into a pro-regeneration macrophage	Via TSG-6/NF-κB/NLRP3 signaling pathway	52
Rat sciatic nerve CCI	Intrathecal administration of 10 μL (1.2 mg/mL) exosomal miR-181c on day 0, day 3 and day 7 after CCI	Attenuated CCI-induced mechanical allodynia and thermal hyperalgesia, decreased IL-6, IL-1β, TNF-α and COX-2	Via modulating neuroinflammation	53
Rat Sciatic nerve transection	20 μg EV in 20 μL PBS were injected into the conduits using a microinjector	Improved the electrophysiology and motor function, improved micro-structure of the regenerated nerves	By increasing the number of newly formed vessels and axonal sprouts	55

### 2.2 *In vitro* studies

A few *in vitro* studies have begun to accumulate on the neuroprotective effects of extracellular vesicles derived from stem cells and their underlying molecular mechanisms, which are listed in Table 2. In this regard, for the first time, Wei and his colleagues investigated the protective effects of exosomes derived from human adipose-derived mesenchymal stem cells and its involved mechanisms in glutamate-induced neuron toxicity.^57^ Results of the study indicated that exosomes have protective effects against glutamate-induced neuron damage, probably via the PI3/K-Akt signaling pathway. In contrast, the study conducted by Zhou et al found that extracellular vesicles derived from mesenchymal stem cells through the ERK pathway activation inhibit RSC96 Schwann cell proliferation and migration as well as promote RSC96 Schwann cell apoptosis,^58^ which may affect Schwann cell-mediated nerve regeneration. Thereafter, results of another study revealed that exosomes from human adipose-derived stem cells via upregulating corresponding genes significantly promote *in vitro* proliferation, migration, and neurotrophic factor secretion of Schwann cells.^29^ Coculture of Schwann cells taken from the site of injured sciatic nerve with adipose-derived stem cells (ADSCs) and ADSC-derived exosomes (ADSC-Exo) at different concentrations reduced Schwann cells apoptosis through upregulation of Bcl-2 and downregulation of Bax mRNA expression.^32^ Recently, it was found that extracellular vesicles secreted by skin precursor-derived Schwann cells could improve neurite outgrowth of dorsal root ganglions sensory neurons in oxygen-glucose-deprivation status, so that these effects have been attributed to miR-21–5p contained in the small extracellular vesicles and miR-21–5p/PTEN/PI3K/Akt axis.^59^ Another study demonstrated for the first time that human umbilical cord mesenchymal stem cell-derived extracellular vesicles (hUCMSC-EVs) through the PI3K/AKT signaling pathway promoted Schwann cells proliferation.^60^ In addition, this study revealed that hUCMSC-EVs through miR-21 transfer mediated the proliferation of Schwann cells. Recently, it has been documented that adipose mesenchymal stem cell-derived exosomes carrying miRNA-22–3p promote Schwann cell proliferation and migration as well as dorsal root ganglion axon growth through downregulation of phosphatase and tensin homolog deleted on chromosome 10 (PTEN).^61^

**Table 2:** Summary of the neuroprotective properties of EVs derived from stem cells *in vitro*.

Origin of vesicles	Induction models	Findings	Involved mechanisms	References
Human adipose-derived mesenchymal stem cells	Neuron damage induced by glutamate	Increased cell survival rates	Activating PI3/K-Akt signaling pathway	57
Rat adipose-derived stem cells	Sciatic nerve injury	Promoted proliferation of Schwann cells and decrease Schwann cells apoptosis	Through Bcl-2 upregulation and Bax downregulation	32
Skin precursor-derived Schwann cells	Oxygen-glucose deprivation	Improved the neurite outgrowth of neurons, enhanced the survival of neurons	Through miR-21–5p/PTEN/PI3K/Akt axis	59

### 2.3 Clinical trial studies

Despite the existence of several studies demonstrating the benefits of extracellular vesicles derived from stem cells as a cell-free treatment in experimental models of nerve injury, the application of this technology in clinical trial studies continue to be elusive and no reliable report has been presented in this field.

## 3 Conclusion

Currently, stem cell-derived extracellular vesicles have attracted the attention of researchers as a cell-free therapy due to their promising biological properties. The neuroprotective properties of extracellular vesicles derived from stem cells as a therapeutic option have been confirmed due to the results of several *in vivo* and *in vitro* preclinical studies. In this regard, it is mentioned that these beneficial effects are mainly caused by anti-inflammatory and anti-apoptotic effects, autophagy reduction, optimization of Schwann cell function, and regulation of regeneration-related genes. The results of these researches indicated that, in general, there is no significant difference between the use of the cells or their extracellular vesicles regarding the amount of nerve regeneration, although in some cases extracellular vesicles had better therapeutic effects. At the same time, delivery by non-invasive methods, crossing the blood–brain barrier and lack of tumorigenicity are among the advantages of extracellular vesicles derived from stem cells compared to stem cells themselves. However, additional preclinical studies are necessary to directly compare the efficacy of different sources and therapeutic doses of EVs, as well as different miRNAs-loaded EVs in another nerve injury models to reach a final accepted protocol with maximum benefits. On the other hand, due to the lack of data demonstrating the neuroprotective effects of stem cell-derived extracellular vesicles in human nerve injury, the design of clinical trial studies on the neuroprotective effects of extracellular vesicles and its possible mechanisms as well as the course of treatment and dose determination is needed. Because generalizing experimental studies to humans requires precision in the differences between animal and human models, such as disease characteristics, drug administration time, and drug efficacy. Another important point is the use of autologous or allogeneic sources of these vesicles, which requires more accuracy and investigation. Therefore, it may be because of these unclear and unresolved issues that clinical use of extracellular vesicles has not yet been reported.

## Financial support and sponsorship

Nil.

## Declaration of competing interest

The authors declare that they have no known competing financial interests or personal relationships that could have appeared to influence the work reported in this paper.

## References

[1] Modrak M., Talukder M.H., Gurgenashvili K., Noble M., Elfar J.C. (2020). Peripheral nerve injury and myelination: potential therapeutic strategies. J Neurosci Res.

[2] Sunderland S. (1951). A classification of peripheral nerve injuries producing loss of function. Brain.

[3] Campbell W.W. (2008). Evaluation and management of peripheral nerve injury. Clin Neurophysiol.

[4] Sulaiman W., Gordon T. (2013). Neurobiology of peripheral nerve injury, regeneration, and functional recovery: from bench top research to bedside application. Ochsner J.

[5] Faroni A., Mobasseri S.A., Kingham P.J., Reid A.J. (2015). Peripheral nerve regeneration: experimental strategies and future perspectives. Adv Drug Deliv Rev.

[6] Magnaghi V., Procacci P., Tata A.M. (2009). Novel pharmacological approaches to Schwann cells as neuroprotective agents for peripheral nerve regeneration. Int Rev Neurobiol.

[7] Sullivan R., Dailey T., Duncan K., Abel N., Borlongan C.V. (2016). Peripheral nerve injury: stem cell therapy and peripheral nerve transfer. Int J Mol Sci.

[8] Currenti S.A. (2010). Understanding and determining the etiology of autism. Cell Mol Neurobiol.

[9] Rossignol D.A., Frye R.E. (2012). A review of research trends in physiological abnormalities in autism spectrum disorders: immune dysregulation, inflammation, oxidative stress, mitochondrial dysfunction and environmental toxicant exposures. Mol Psychiatr.

[10] Fu S.Y., Gordon T. (1995). Contributing factors to poor functional recovery after delayed nerve repair: prolonged axotomy. J Neurosci.

[11] You S., Petrov T., Chung P.H., Gordon T. (1997). The expression of the low affinity nerve growth factor receptor in long-term denervated Schwann cells. Glia.

[12] Guest J.D., Rao A., Olson L., Bunge M.B., Bunge R.P. (1997). The ability of human Schwann cell grafts to promote regeneration in the transected nude rat spinal cord. Exp Neurol.

[13] Jiang L., Jones S., Jia X. (2017). Stem cell transplantation for peripheral nerve regeneration: current options and opportunities. Int J Mol Sci.

[14] Orbay H., Uysal A.C., Hyakusoku H., Mizuno H. (2012). Differentiated and undifferentiated adipose-derived stem cells improve function in rats with peripheral nerve gaps. J Plast Reconstr Aesthetic Surg.

[15] Tohill M., Terenghi G. (2004). Stem-cell plasticity and therapy for injuries of the peripheral nervous system. Biotechnol Appl Biochem.

[16] Ribeiro-Resende V., Pimentel-Coelho P., Mesentier-Louro L. (2009). Trophic activity derived from bone marrow mononuclear cells increases peripheral nerve regeneration by acting on both neuronal and glial cell populations. Neuroscience.

[17] Sowa Y., Imura T., Numajiri T., Nishino K., Fushiki S. (2012). Adipose-derived stem cells produce factors enhancing peripheral nerve regeneration: influence of age and anatomic site of origin. Stem Cell Dev.

[18] Essa M.M., Qoronfleh M.W. (2020).

[19] Gnecchi M., He H., Liang O.D. (2005). Paracrine action accounts for marked protection of ischemic heart by Akt-modified mesenchymal stem cells. Nat Med.

[20] Takahashi M., Li T.-S., Suzuki R. (2006). Cytokines produced by bone marrow cells can contribute to functional improvement of the infarcted heart by protecting cardiomyocytes from ischemic injury. Am J Physiol Heart Circ Physiol.

[21] Isele N.B., Lee H.-S., Landshamer S. (2007). Bone marrow stromal cells mediate protection through stimulation of PI3-K/Akt and MAPK signaling in neurons. Neurochem Int.

[22] Timmers L., Lim S.K., Hoefer I.E. (2011). Human mesenchymal stem cell-conditioned medium improves cardiac function following myocardial infarction. Stem Cell Res.

[23] Raposo G., Stoorvogel W. (2013). Extracellular vesicles: exosomes, microvesicles, and friends. J Cell Biol.

[24] Kalani A., Tyagi A., Tyagi N. (2014). Exosomes: mediators of neurodegeneration, neuroprotection and therapeutics. Mol Neurobiol.

[25] Andaloussi S.E., Lakhal S., Mäger I., Wood M.J. (2013). Exosomes for targeted siRNA delivery across biological barriers. Adv Drug Deliv Rev.

[26] Gimona M., Pachler K., Laner-Plamberger S., Schallmoser K., Rohde E. (2017). Manufacturing of human extracellular vesicle-based therapeutics for clinical use. Int J Mol Sci.

[27] Lener T., Gimona M., Aigner L. (2015). Applying extracellular vesicles based therapeutics in clinical trials–an ISEV position paper. J Extracell Vesicles.

[28] Vogel A., Upadhya R., Shetty A.K. (2018). Neural stem cell derived extracellular vesicles: attributes and prospects for treating neurodegenerative disorders. EBioMedicine.

[29] Chen J., Ren S., Duscher D. (2019). Exosomes from human adipose-derived stem cells promote sciatic nerve regeneration via optimizing Schwann cell function. J Cell Physiol.

[30] Yin G., Yu B., Liu C. (2021). Exosomes produced by adipose-derived stem cells inhibit Schwann cells autophagy and promote the regeneration of the myelin sheath. Int J Biochem Cell Biol.

[31] Ma Y., Dong L., Zhou D. (2019). Extracellular vesicles from human umbilical cord mesenchymal stem cells improve nerve regeneration after sciatic nerve transection in rats. J Cell Mol Med.

[32] Liu C.Y., Yin G., Sun Y.D. (2020). Effect of exosomes from adipose-derived stem cells on the apoptosis of Schwann cells in peripheral nerve injury. CNS Neurosci Ther.

[33] Zhao J., Ding Y., He R. (2020). Dose-effect relationship and molecular mechanism by which BMSC-derived exosomes promote peripheral nerve regeneration after crush injury. Stem Cell Res Ther.

[34] Ma Y., Ge S., Zhang J. (2017). Mesenchymal stem cell-derived extracellular vesicles promote nerve regeneration after sciatic nerve crush injury in rats. Int J Clin Exp Pathol.

[35] Bucan V., Vaslaitis D., Peck C.-T., Strauß S., Vogt P.M., Radtke C. (2019). Effect of exosomes from rat adipose-derived mesenchymal stem cells on neurite outgrowth and sciatic nerve regeneration after crush injury. Mol Neurobiol.

[36] Xia B., Gao J., Li S. (2019). Extracellular vesicles derived from olfactory ensheathing cells promote peripheral nerve regeneration in rats. Front Cell Neurosci.

[37] Horwitz E., Dominici M. (2008). How do mesenchymal stromal cells exert their therapeutic benefit?. Cytotherapy.

[38] Costigan M., Scholz J., Woolf C.J. (2009). Neuropathic pain: a maladaptive response of the nervous system to damage. Annu Rev Neurosci.

[39] Shiue S.-J., Rau R.-H., Shiue H.-S. (2019). Mesenchymal stem cell exosomes as a cell-free therapy for nerve injury–induced pain in rats. Pain.

[40] Mao Q., Nguyen P.D., Shanti R.M. (2019). Gingiva-derived mesenchymal stem cell-extracellular vesicles activate Schwann cell repair phenotype and promote nerve regeneration. Tissue Eng.

[41] Rao F., Zhang D., Fang T. (2019). Exosomes from human gingiva-derived mesenchymal stem cells combined with biodegradable chitin conduits promote rat sciatic nerve regeneration. Stem Cell Int.

[42] Zhang Y., Wang W.-T., Gong C.-R., Li C., Shi M. (2020). Combination of olfactory ensheathing cells and human umbilical cord mesenchymal stem cell-derived exosomes promotes sciatic nerve regeneration. Neural Regen Res.

[43] Chen X., Ye K., Yu J. (2020). Regeneration of sciatic nerves by transplanted microvesicles of human neural stem cells derived from embryonic stem cells. Cell Tissue Bank.

[44] Hsu J.-M., Shiue S.-J., Yang K.D. (2020). Locally applied stem cell exosome-scaffold attenuates nerve injury-induced pain in rats. J Pain Res.

[45] Kuo P.-J., Rau C.-S., Wu S.-C. (2021). Exosomes secreted by adipose-derived stem cells following FK506 stimulation reduce autophagy of macrophages in spine after nerve crush injury. Int J Mol Sci.

[46] Rau C.-S., Kuo P.-J., Wu S.-C. (2021). Enhanced nerve regeneration by exosomes secreted by adipose-derived stem cells with or without FK506 stimulation. Int J Mol Sci.

[47] Fan B., Chopp M., Zhang Z.G., Liu X.S. (2021). Treatment of diabetic peripheral neuropathy with engineered mesenchymal stromal cell-derived exosomes enriched with microRNA-146a provide amplified therapeutic efficacy. Exp Neurol.

[48] Yang Z., Yang Y., Xu Y. (2021). Biomimetic nerve guidance conduit containing engineered exosomes of adipose-derived stem cells promotes peripheral nerve regeneration. Stem Cell Res Ther.

[49] Li S., Huang C., Tu C. (2022). Bone marrow mesenchymal stem cell-derived exosomes shuttling miR-150-5p alleviates mechanical allodynia in rats by targeting NOTCH2 in microglia. Mol Med.

[50] Lu Y., Zhang J., Zeng F. (2022). Human PMSCs-derived small extracellular vesicles alleviate neuropathic pain through miR-26a-5p/Wnt5a in SNI mice model. J Neuroinflammation.

[51] Demyanenko S.V., Pitinova M.A., Kalyuzhnaya Y.N. (2022). Human multipotent mesenchymal stromal cell–derived extracellular vesicles enhance neuroregeneration in a rat model of sciatic nerve crush injury. Int J Mol Sci.

[52] Li C., Li X., Shi Z. (2022). Exosomes from LPS-preconditioned bone marrow MSCs accelerated peripheral nerve regeneration via M2 macrophage polarization: involvement of TSG-6/NF-κB/NLRP3 signaling pathway. Exp Neurol.

[53] Zhang Y., Ye G., Zhao J. (2022). Exosomes carried miR-181c-5p alleviates neuropathic pain in CCI rat models. An Acad Bras Ciências.

[54] Chen S.-H., Kao H.-K., Wun J.-R. (2022). Thermosensitive hydrogel carrying extracellular vesicles from adipose-derived stem cells promotes peripheral nerve regeneration after microsurgical repair. APL bioengineering.

[55] Zhang W., Fang X.-X., Li Q.-C., Pi W., Han N. (2023). Reduced graphene oxide-embedded nerve conduits loaded with bone marrow mesenchymal stem cell-derived extracellular vesicles promote peripheral nerve regeneration. Neural Regeneration Research.

[56] Zhu Z., Zhang Y., Huang Z., Hao H., Yan M. (2023). Hypoxic culture of umbilical cord mesenchymal stem cell-derived sEVs prompts peripheral nerve injury repair. Front Cell Neurosci.

[57] Wei J., Chen Y., Xue C. (2016). Protection of nerve injury with Exosome extracted from mesenchymal stem cell. Zhongguo Yi Xue Ke Xue Yuan Xue Bao.

[58] Zhou D., Zhai W., Zhang M. (2018). Mesenchymal stem cell-derived extracellular vesicles promote apoptosis in rsc96 Schwann cells through the activation of the erk pathway. Int J Clin Exp Pathol.

[59] Cong M., Shen M., Wu X. (2021). Improvement of sensory neuron growth and survival via negatively regulating PTEN by miR-21-5p-contained small extracellular vesicles from skin precursor-derived Schwann cells. Stem Cell Res Ther.

[60] Ma Y., Zhou D., Zhang H., Tang L., Qian F., Su J. (2021). Human umbilical cord mesenchymal stem cell-derived extracellular vesicles promote the proliferation of schwann cells by regulating the PI3K/AKT signaling pathway via transferring miR-21. Stem Cell Int.

[61] Yang J., Wang B., Wang Y. (2022). Exosomes derived from adipose mesenchymal stem cells carrying miRNA-22-3p promote schwann cells proliferation and migration through downregulation of PTEN. Dis Markers.

